# Serum complexed and free prostate specific antigen levels are lower in female elite athletes in comparison to control women

**DOI:** 10.12688/f1000research.11821.1

**Published:** 2017-07-17

**Authors:** Emma Eklund, Eleftherios P Diamandis, Carla Muytjens, Sarah Wheeler, Anu Mathew, Martin Stengelin, Eli Glezer, Galina Nikolenko, Marshall D. Brown, Yingye Zheng, Angelica Lindén Hirschberg

**Affiliations:** 1 Department of Women’s and Children’s Health, Division of Obstetrics and Gynecology, Karolinska Institutet, Stockholm, Sweden; 2 Department of Laboratory Medicine and Pathobiology, University of Toronto, Toronto, Ontario, Canada; 3 Department of Clinical Biochemistry, University Health Network, Toronto, Ontario, Canada; 4 Department of Pathology and Laboratory Medicine, Mount Sinai Hospital, Toronto, Ontario, Canada; 5 Meso Scale Diagnostics, LLC. , Rockville, MD, USA; 6 Department of Biostatistics, Fred Hutchinson Cancer Research Center, Seattle, WA, USA; 7 Department of Gynecology and Reproductive Medicine, Karolinska University Hospital, Stockholm, Sweden

**Keywords:** prostate specific antigen, elite female athletes, hyperandrogenism, fifth-generation PSA assays, serum PSA in women, Olympic teams

## Abstract

Background: We hypothesize that prostate specific antigen (PSA), a protein that it is under regulation by androgens, may be differentially expressed in female elite athletes in comparison to control women.

Methods: We conducted a cross-sectional study of 106 female athletes and 114 sedentary age-matched controls.  Serum from these women was analyzed for complexed prostate specific antigen (cPSA) and free prostate specific antigen (fPSA), by fifth generation assays with limits of detection of around 6 and 140 fg/mL, respectively.  A panel of estrogens, androgens and progesterone in the same serum was also quantified by tandem mass spectrometry.

Results: Both components of serum PSA (cPSA and fPSA) were lower in the elite athletes vs the control group (P=0.033 and 0.013, respectively).  Furthermore, estrone (p=0.003) and estradiol (p=0.004) were significantly lower, and dehydroepiandrosterone  (p=0.095) and 5-androstene-3β, 17β-diol (p=0.084) tended to be higher in the athletes vs controls. Oral contraceptive use was similar between groups and significantly associated with increased cPSA and fPSA in athletes (p= 0.046 and 0.009, respectively).  PSA fractions were not significantly associated with progesterone changes. The Spearman correlation between cPSA and fPSA in both athletes and controls was 0.75 (P < 0.0001) and 0.64 (P < 0.0001), respectively.

Conclusions: Elite athletes have lower complexed and free PSA, higher levels of androgen precursors and lower levels of estrogen in their serum than sedentary control women.

Abbreviations: cPSA, complexed PSA; fPSA, free PSA; PCOS, polycystic ovarian syndrome; E1, estrone; E2, estradiol; DHEA, dehydroepiandrosterone, Testo, testosterone; DHT, dihydrotestosterone; PROG, progesterone; Delta 4, androstenedione; Delta 5, androst-5-ene-3β, 17β-diol; BMD, body mineral density; LLOQ, lower limit of quantification; ULOQ, upper limit of quantification; LOD, limit of detection; ACT, α _1_-antichymotrypsin

## Introduction

Prostate specific antigen (PSA) is a well-known and clinically useful biomarker of prostate adenocarcinoma ^1^. PSA circulates in blood of males as a complex with alpha 1 antichymotrypsin (cPSA) (approx. 80% of total) or as free, non-complexed PSA (fPSA) (approx. 20% of total) ^2, 3^. It has now been well-documented that PSA is also produced by many female tissues, including breast, periurethral, salivary and thyroid tissues, and by many tumors ^4, 5^. The PSA gene is up-regulated by androgens and progestins in breast and other female tissues, as well as in model systems such as breast carcinoma cell lines ^6– 12^. Serum PSA in women fluctuates during the menstrual cycle, and these changes are attributed to up-regulation by progesterone during the luteal phase ^13– 15^.

PSA also circulates in female serum as complexed and free PSA ^16^, but its concentrations are exceedingly low (around 1pg/mL), precluding accurate determination with third generation PSA assays ^17, 18^. In some circumstances, such as in women with hyperandrogenic syndromes, including polycystic ovarian syndrome (PCOS) and hirsutism, it has been shown that total PSA in female serum is elevated and this finding may be used as an aid to disease diagnosis ^19– 25^.

Recently, fifth generation PSA assays have been developed by many groups, allowing accurate PSA determinations in the low fg/mL range ^26– 31^. These assays can quantify both complexed and free PSA in serum and urine of females and allow examination of the possible role of female PSA in healthy and disease states. We recently confirmed the diagnostic value of cPSA and fPSA in women with PCOS by using one of these assays ^32^.

It has previously been speculated that female elite athletes may have higher circulating androgen levels than sedentary age-matched females, and that hyperandrogenic syndromes such as PCOS, congenital adrenal hyperplasia and 46XY disorders of sex development are more common in elite athletes ^33– 37^.

In this paper we speculated that serum cPSA and fPSA, due to their in-vivo up-regulation by androgens and progestins, as seen with female-to-male transsexuals treated with testosterone ^38, 39^, may represent an integrated index of total androgenic/progestational activity in female tissues. We thus examine here, the levels of serum complexed and free PSA in 106 female elite athletes and 114 age-matched sedentary controls, along with levels of estrogens, androgens and progesterone. The observed differences in serum cPSA and fPSA between athletes and controls were examined in the context of oral contraceptive use and ovulatory cycles.

## Materials and methods

### Subjects

This project was approved by the Regional Ethics Committee of the Karolinska Institutet, Stockholm, Sweden (EPN 2011-1426-3). Serum samples from 106 Swedish Olympic female athletes representative of the Swedish participation in the summer and winter Olympic games, were recruited in connection with pre-Olympic training camps. Participants were at least 18 years of age. Samples were also collected from 114 age-matched, healthy non-athletic female controls (maximum of 2 hours endurance and/or strength training per week and no prior participation in elite level competition). Recruitment started in November, 2011 and was completed by April, 2015.

The subjects were investigated at the Women’s Health Research Unit, Karolinska University Hospital or in connection with training camps. Data on ethnicity, past and present health problems, injuries, medications, gynecological history (bleeding pattern, date of last menstruation, pregnancies, hormonal contraceptive use) and symptoms of hyperandrogenism (hirsutism, acne), were collected by a general health questionnaire. Furthermore, data on sport discipline, training hours per week, achieved sport performance and goals were collected from the Olympic athletes. A fasting blood sample was collected by a standard venipuncture between 7–9AM. Body composition (body fat, muscle mass, BMD) was investigated by dual X-ray absorptiometry (DXA), at the Department of Radiology, Karolinska University Hospital, Solna, Stockholm.

Serum from both athletes and controls was analyzed by tandem mass spectrometry for the following steroid hormones and metabolites: estrone (E1), estradiol (E2), testosterone (Testo), dehydroepiandrosterone (DHEA), androstenedione (Delta 4), androst-5-ene-3β, 17β-diol (Delta 5), dihydrotestosterone (DHT), and progesterone (PROG).

Serum samples were kept frozen at -80°C until thawed for analysis. Details of the tandem mass spectrometry analysis have been described elsewhere ^40^. A progesterone concentration ≥ 5.3 ng/mL in the luteal phase of the menstrual cycle was taken as an indication of successful ovulation.

Among the athletes, 65 were not using contraceptives and 41 were using hormonal contraceptives (39%). Among the control group, 69 were not using contraceptives and 45 were using contraceptives (39%). The type of oral contraceptive used was quite variable and included the following combinations: Ethinylestradiol + Levonorgestrel, Ethinylestradiol + Etonogestrel, Ethinylestradiol + Drospirenon, Ethinylestradiol + Dienogest, Ethinylestradiol + Norgestimate, Ethinylestradiol + Cyproterone, Ethinylestradiol + Nomegestrol, Ethinylestradiol + Noretisterone, or the following single progestins: Desogestrel, Etonogestrel, Levonorgestrel. Due to the small number of participants, we did not analyze the data according to type of contraceptive used.

### Measurement of complexed and free PSA

One vial of 200µL serum per sample was provided blinded to Meso Scale Diagnostics (MSD) for cPSA and fPSA measurement using MSD’s MULTI-ARRAY® electrochemiluminescence technology in the S-PLEX ^TM^ format, which allows quantitation of previously unmeasurable levels of biomarkers with fg/mL sensitivity ^27, 41^. The samples were thawed and centrifuged at 10,000g for 10 minutes at 4˚C before being aliquoted into low retention 96-well round bottom plates for subsequent testing. Plates were immediately frozen on dry ice and stored at -80˚C until testing. cPSA and fPSA assays were calibrated to the WHO International Standard for prostate-specific antigen, with 90% bound to alpha _1_-antichymotrypsin and 10% in the free form (National Institute for Biological Standards and Control, [NIBSC], code 96/670, Hertfordshire, England) and the WHO International Standard for prostate-specific antigen free (NIBSC, code 96/668, Hertfordshire, England), respectively. Assay characteristics were determined prior to sample testing.

For each assay, 8-point calibration curves were included on each plate, and the data were fitted with a weighted 4-parameter logistic curve fit. Limit of detection (LOD) is a calculated concentration corresponding to the average signal 2.5 standard deviations above the background (zero calibrator). Lower limit of quantitation (LLOQ) and upper limit of quantitation (ULOQ) were established for the plate lot by measuring multiple levels of calibrator near the expected LLOQ and ULOQ. LLOQ and ULOQ are, respectively, the lowest and highest concentration of calibrator tested which has a %CV of 20% or less, with recovered concentration within 70–130%. The LOD was 5.7 and 140 fg/mL for cPSA and fPSA assays, respectively. The LLOQ was 17 and 480 fg/mL for cPSA and fPSA assays, respectively. The ULOQ was 72,000 and 240,0000 fg/mL for cPSA and fPSA assays, respectively. Precision was determined from testing of three internal quality control samples that span the detectable range and is expressed as the % CV from 16 specimen assay runs, with 2 operators over 3 testing days. % CVs were between 11–13% for cPSA and 6–23% for fPSA.

Serum, EDTA plasma, and heparin plasma samples (7–8 samples total) were spiked with calibrator at two or three concentrations. The non-complexing form of PSA (Scripps Laboratories, San Diego, CA; #90024) that does not bind to a1- antichymotrypsin (ACT) was used in spike recovery experiments for the fPSA assay. Average spike recoveries for the fPSA and cPSA assays were 88% and 90%, respectively. Serum, EDTA plasma, and heparin plasma samples (7–8 samples total) were diluted 2, 4 and 8-fold. Average dilution linearities for the fPSA and cPSA assays were 114% and 109%, respectively.

The samples and calibrator dilutions were assayed in duplicate and all samples were measured for cPSA and fPSA. Measurement of cPSA was performed with a 2-fold dilution of the samples; fPSA measurement was performed on neat samples. Concentrations of biomarkers in each sample were calculated from the calibrator curves taking into account sample dilutions. The mean of two measurements was derived for each analyte in each sample and reported in fg/mL.

### Statistical analysis

We first categorized the athletes and control subjects by several clinical and demographic variables and hormonal measurements. When comparing variables among athletes and controls, the Wilcoxon rank sum test was used to determine if there were significant differences. Correlation of parameters between athletes and controls were examined using the Spearman correlation coefficient.

Lower limits of detection (LOD) for cPSA and fPSA assays are 6 fg/mL and 140 fg/mL, respectively. Since measurements that fall below these values are unreliable, we set marker measurements that fall below the LOD to LOD/2. Among all samples, 102/206 serum fPSA values but none of the serum cPSA values were below the LOD of the method used. Similarly, 110 out of 220 progesterone values were below the detection limit of the method (0.10 ng/mL) and these values were adjusted to 0.05 ng/mL. Rank-based non-parametric methods were employed so that the results would be robust to these transformations.

## Results

The included excel file contains all anthopometric and biochemical data used to perform statistical analyses. Significant differences between athletes and controls, irrespective of oral contraceptive use, were noted for the following variables: total BMD, spine BMD, lean mass (all parameters were elevated in athletes; p<0.001) and fat percentage (decreased in athletes; p<0.001).

Initial examination of our data revealed the significant effect of oral contraceptives on hormonal and PSA measurements. For this reason, we stratified both athlete and control groups by hormonal measurements and PSA, according to oral contraceptive use (data shown in Table 1 and Table 2). E1 and E2 levels were significantly reduced in athletes (p=0.003 and 0.004, respectively). Other measurements were not significantly different between athletes and controls that were not taking oral contraceptives, but there was a trend for athletes to have higher DHEA (p=0.095) and Delta 5 (p=0.084). In Table 2 it is shown that both cPSA and fPSA were significantly lower in athletes in comparison to control subjects, irrespective of hormonal contraceptive use. Median cPSA was 776 fg/mL in athletes and 1249 fg/mL in controls (p=0.003). Median fPSA was 70 fg/mL in athletes and 169.5 fg/mL in controls (p=0.013).

**Table 1.** Median (25th, 75th percentiles) anthropometric measurements of athletes and controls, stratified by hormonal contraceptive use.

	Athletes (n = 106)				Controls (n = 114)			
	No Hormonal Contraceptives		Hormonal Contraceptives		No Hormonal Contraceptives		Hormonal Contraceptives	
	n ^2^		n		n		n	
Age (years)	65	26 (22, 30)	41	24 (22, 27)	69	25 (23, 30)	45	24 (22, 26)
Weight (kg)	64	65 (60, 70.2)	41	62 (60, 68.7)	69	60 (56, 68)	45	62 (55.5, 68)
Total BMD (g/cm3)	41	1.25 (1.19, 1.31)	24	1.23 (1.16, 1.31)	59	1.15 (1.08, 1.2)	39	1.13 (1.1, 1.18)
Spine BMD (g/cm)	41	1.12 (1.01, 1.19)	24	1.1 (1.03, 1.19)	59	1.02 (0.94, 1.07)	39	0.99 (0.95, 1.03)
Fat %	41	17.2 (13.3, 22.6)	24	19.7 (15.6, 21.6)	59	31 (27.2, 35.5)	39	32.1 (26.9, 36.6)
thorax/total (fat)	40	0.45 (0.42, 0.47)	24	0.43 (0.42, 0.46)	59	0.45 (0.41, 0.49)	39	0.44 (0.41, 0.48)
bone/total (fat)	40	0.43 (0.41, 0.45)	24	0.45 (0.41, 0.46)	59	0.42 (0.4, 0.46)	39	0.43 (0.41, 0.46)
lean mass total (kg)	41	50.2 (46.4, 53.2)	24	48.9 (47.1, 52.6)	59	39.5 (37.7, 42.8)	39	40.6 (37.3, 43.6)
lean mass legs (kg)	41	17.7 (15.5, 18.6)	24	17.4 (16.3, 18.8)	59	13.6 (12.5, 14.3)	39	13.8 (12.4, 15.3)
total mass (kg)	41	64.2 (59.5, 67.8)	24	63.6 (60.5, 70.9)	59	61.1 (55.7, 66.6)	39	63.9 (55.9, 69.5)
E1 (pg/mL) ^1^	65	43.4 (34, 65.5)	41	23 (16.3, 33.8)	69	60.1 (40.5, 89.4)	45	23.9 (18.9, 34.1)
E2 (pg/mL)	65	67 (32.3, 106.5)	41	13.1 (4.2, 30)	69	109.9 (45.7, 161.9)	45	10.1 (3.6, 33.4)
DHEA (ng/mL)	65	7.7 (5.4, 10.9)	41	6.5 (4.1, 9.8)	69	6.1 (4.7, 8.9)	45	5 (4, 5.9)
Testo (pg/mL)	65	280 (218, 378)	41	235 (166, 345)	69	282 (242, 348)	45	265 (185, 314)
Delta4 (pg/mL)	65	1411 (1069, 1577)	41	987 (755, 1236)	69	1379 (1063, 1765)	45	891 (711, 1113)
Delta5 (pg/mL)	65	717 (529, 930)	40	672 (458, 826)	69	619 (479, 801)	45	572 (435, 731)
DHT (pg/mL)	65	107.6 (79.6, 138.3)	41	98.6 (74.6, 121.4)	69	103.4 (87.8, 168.7)	45	97.8 (77.5, 140.4)
PROG (ng/mL)	65	0.16 (0.11, 1.19)	41	0.05 (0.05, 0.11)	69	0.13 (0.05, 7.18)	45	0.05 (0.05, 0.05)
PROG > 5.3 ng/mL	65	10 (15%)	41	1 (2%)	69	19 (28%)	45	0 (0%)
cPSA (serum), fg/mL	63	776 (353.5, 2165)	40	1812 (579, 3236)	68	1249 (509.8, 3985.5)	45	2002 (690, 2369)
fPSA (serum), fg/mL	63	70 (70, 216.5)	40	216.5 (70, 525.2)	68	169.5 (70, 394.8)	45	220 (70, 379)

^1^See abbreviation list for full details ^2^Number of participants

**Table 2.** Median (25th, 75th percentiles) steroid and PSA values among athletes and controls who do not use contraceptives.

	Athletes (n = 65)		Controls (n =69)		p-value ^1^
E1 (pg/mL)	43.4	(34, 65.5)	60.1	(40.5, 89.4)	0.003
E2 (pg/mL)	67	(32.3, 106.5)	109.9	(45.7, 161.9)	0.004
DHEA (ng/mL)	7.7	(5.4, 10.9)	6.1	(4.7, 8.9)	0.095
Testo (pg/mL)	280	(218, 378)	282	(242, 348)	0.831
Delta 4 (pg/mL)	1411	(1069, 1577)	1379	(1063, 1765)	0.947
Delta5 (pg/mL)	717	(529, 930)	619	(479, 801)	0.084
DHT (pg/mL)	107.6	(79.6, 138.3)	103.4	(87.8, 168.7)	0.257
PROG (ng/mL)	0.16	(0.11, 1.19)	0.13	(0.05, 7.18)	0.7
PROG ≥ 5.3 ng/mL	10/55 (15%)		19/50 (28%)		0.134
cPSA (serum) fg/mL	776	(353.5, 2165)	1249	(509.8, 3985.5)	0.033
fPSA (serum) fg/mL	70	(70, 216.5)	169.5	(70, 394.8)	0.013

^1^P-values calculated using Wilcoxon signed rank test.

It is shown in Table 3 that in athletes, the use of oral contraceptives significantly increased both cPSA (from 776 to 1812 fg/mL; (p=0.046) and fPSA from 70 to 216 fg/mL (p=0.009). In control subjects, we noticed similar trends (from 1249 fg/mL to 2002 fg/mL for cPSA and from 169.5 fg/mL to 220 fg/mL for fPSA) but the differences were not significant. The elevation of serum PSA with oral contraceptive use in women has been reported before ^9^.

**Table 3.** Comparison of cPSA and fPSA levels between women who take hormonal contraceptives and those who do not, for athletes and controls. Numbers represent medians (25th, 75th percentiles).

	No Hormonal Contraceptives		Hormonal Contraceptives		p-value ^1^
Athletes					
cPSA (fg/mL)	776	(353.5, 2165) ^2^	1812	(579, 3236)	0.04628
fPSA (fg/mL)	70	(70, 216.5)	216.5	(70, 525.2)	0.00917
Controls					
cPSA (fg/mL)	1249	(509.8, 3985.5)	2002	(690, 2369)	0.62432
fPSA (fg/mL)	169.5	(70, 394.8)	220	(70, 379)	0.53344

^1^P-values calculated using Wilcoxon signed rank test. ^2^Numbers represent medians (25 ^th^ – 75 ^th^ percentiles).

The distribution of all clinical and demographic variables between athletes and controls, stratified by oral contraceptive use, is shown in Supplementary Figure 1. The distribution of all hormonal and PSA measurements in controls and athletes, stratified by oral contraceptive use, is shown in Supplementary Figure 2 and Supplementary Figure 3.

**Figure 1.**
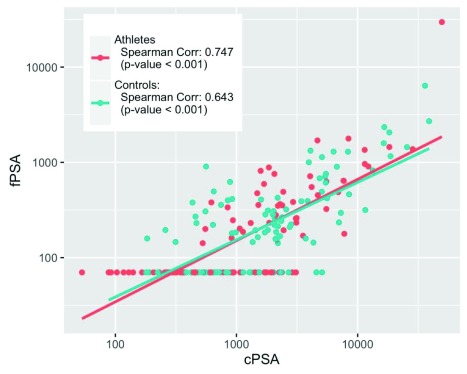
Serum fPSA vs. cPSA in athletes and controls. Spearman correlations (p-values) are shown.

**Figure 2.**
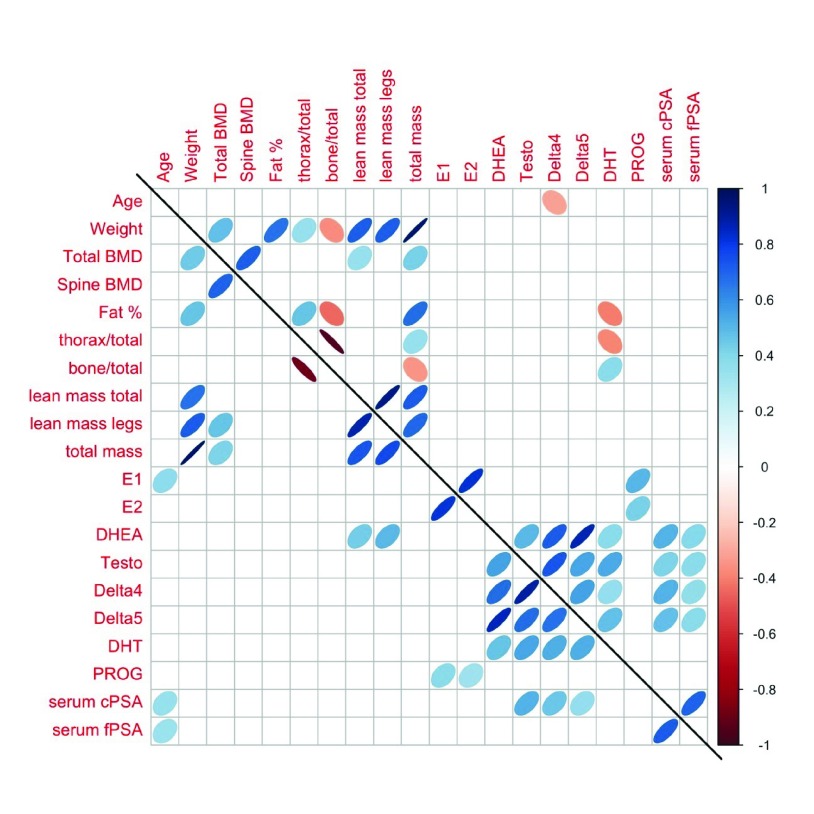
Spearman correlation plots among all variables in athletes (lower diagonal) and controls (upper diagonal) for women not taking hormonal contraceptives. The pairs for which ovals are displayed indicate a Spearman correlation that is significantly different from zero, with p-value < 0.01. See also Table 1 for parameter description.

We then examined the correlation between cPSA and fPSA in serum of both athletes and controls. The Spearman correlation coefficients (rs) were highly significant for both groups of subjects ( Figure 1). For athletes, rs=0.75 (p < 0.001) and for controls, rs=0.64 (p < 0.001). We also examined the correlation between the two PSA variables and all other clinical, demographic and hormonal variables in subjects not taking oral contraceptives ( Supplementary Table 1). The only statistically significant correlations (p < 0.01; all positive) were between cPSA and fPSA and age, and cPSA with Testo, Delta4 and Delta5 in the athletes group, and between cPSA and fPSA with DHEA, Testo, Delta4 and Delta5 in the control group. The correlations between all of the available parameters in the control and athlete groups not taking oral contraceptives are further depicted in Figure 2.

We also examined whether cPSA and fPSA correlated with progesterone. We found no correlation, in either the control or athlete groups, as stratified by hormonal contraceptive use ( Supplementary Figure 4). We further dichotomously classified progesterone as being ≥ 5.3 ng/mL (indicative of luteal phase) or < 5.3 ng/mL in athletes and controls not using oral contraceptives, and examined any association with cPSA or fPSA. We found no association ( Supplementary Figure 5).

Anthropometric and biochemical data for athletes and controls The data file contains all available anthropometric and biochemical data for all athletes and controls included in this study, and includes free PSA and complexed PSA. It was used to do statistics and to derive the results of this paper. Click here for additional data file. Copyright: © 2017 Eklund E et al. 2017 Data associated with the article are available under the terms of the Creative Commons Zero "No rights reserved" data waiver (CC0 1.0 Public domain dedication).

## Discussion

PSA is used widely for diagnosis and monitoring of prostatic adenocarcinoma in males ^1^. Quantification of PSA in female serum is problematic, due to its very low concentrations ^4, 5^. Newly developed fifth generation PSA assays demonstrate enough sensitivity for quantifying both complexed PSA and free PSA in serum of normal women ^27^. We have recently shown that median cPSA concentration in normal women is around 900 fg/mL, while fPSA is about ten times lower (70 fg/mL). In women with PCOS, serum PSA concentration is elevated by about 3-fold ^32^.

It is well-known that the PSA gene is up-regulated by androgens ^7– 12^. Previous studies have shown that women who are taking androgenic steroids to change sex (e.g. young female-to-male transsexuals) demonstrate high elevations of serum PSA and urine PSA ^37, 38^. We have also shown previously that women who are taking oral contraceptives upregulate their PSA at both the tissue and the serum level ^9^. Based on this data, we hypothesized that serum PSA concentration may represent a novel integrated index of androgenic stimulation in normal women.

It has been suggested that elite athletes more frequently suffer from various hyperandrogenic syndromes such as PCOS, congenital adrenal hyperplasia and 46XY disorders of sex development ^33– 37^. In this paper, we examine the possible differences in serum PSA between Olympic elite athletes and sedentary control women. For this, we measured both cPSA and fPSA, as well as a panel of androgens, estrogens, and progesterone in serum.

We have shown that in athletes, E1 and E2 concentrations are significantly reduced, whereas levels of DHEA and Delta 5 tend to be slightly elevated. Moreover, we identified for the first time a significant decrease in serum cPSA and fPSA in elite athletes, in comparison to the control group. We have also confirmed the previous finding ^9^ that oral contraceptive use increases both cPSA and fPSA in serum of athletes (but the differences were not significant in the control group, although the same trend was observed).

Recently, we speculated that serum and urine PSA in female athletes could be used to test for doping with androgenic steroids ^42^. In this work, we did not examine this possibility due to lack of samples from provenly doping athletes. However, now that serum PSA in women can be reliably quantified, this parameter could be considered for inclusion into the athlete’s biological passport, as suggested by Sottas *et al* ^43^.

Our finding that serum E1 and E2 are lower in athletes or exercising individuals in comparison to controls is supported by previous literature ^43– 45^. An explanation for this phenomenon may be related to menstrual cycle disturbances induced by strenuous exercise and stress during the competition. Such findings have prompted some to speculate that the lower incidence of breast cancer in individuals who exercise may be due to reduced estrogen and androgen levels ^43– 45^. Previously, we also established a connection between tumoral or nipple aspirate PSA and breast cancer prognosis ^46– 49^. Clearly, the interconnections between exercise, androgen, estrogen and serum PSA levels and breast cancer need to be better defined.

It has been reported before that serum PSA fluctuates with the menstrual cycle, likely due to up-regulation by progesterone in the luteal phase ^13– 15^. We examined the correlation and the association of cPSA and fPSA with serum progesterone, as a continuous and dichotomous variable, respectively. Our results have shown that there was no correlation or association between either cPSA or fPSA and progesterone or with the frequency of ovulatory cycles, (ovulation being defined as progesterone ≥ 5.4 ng/mL in the luteal phase).

While we found that many anthropometric measurements are different between athletes and controls ( Table 1), among steroid measurements, only E1, E2, and to a lesser extent DHEA and Delta5 concentrations were different between the two groups. However, we identified a significant difference between both cPSA and fPSA between elite athletes and controls. The differences in cPSA and fPSA levels between these two groups do not seem to be associated with hyperandrogenism or the menstrual cycle. It will be interesting to examine, in the future, what other parameters are responsible for the differential concentrations of cPSA and fPSA in serum of these women. Since PSA in women originates mostly from the breast, differences in breast size, which was one parameter we did not study, could be one possible cause for the different levels of these proteins.

## Data availability

The data referenced by this article are under copyright with the following copyright statement: Copyright: © 2017 Eklund E et al.

Data associated with the article are available under the terms of the Creative Commons Zero "No rights reserved" data waiver (CC0 1.0 Public domain dedication). 

**Dataset 1: Anthropometric and biochemical data for athletes and controls.** The data file contains all available anthropometric and biochemical data for all athletes and controls included in this study, and includes free PSA and complexed PSA. It was used to do statistics and to derive the results of this paper. DOI, 10.5256/f1000research.11821.d168039 ^50^

## Consent

This study was approved by the Regional Ethics Committee of the Karolinska Institutet, Stockholm, Sweden (EPN 2011-1426-3). Written informed consent was obtained from all participants.

## Supplementary material

**Supplementary Figure 1**: Distribution of age, weight and body composition variables between athletes and controls, stratified by oral contraceptive use.

**Supplementary Figure 2:** Distributions of steroid hormone variables among athletes and controls, stratified by oral contraceptive use.

**Supplementary Figure 3:** Distributions of serum PSA measurements among athletes and controls, stratified by oral contraceptive use.

**Supplementary Figure 4:** Correlation between PSA values and progesterone in athletes and controls, stratified by hormonal contraceptive use. No correlation was found.

**Supplementary Figure 5:** PSA values stratified by progesterone < 5.3 vs. ≥ 5.4 ng/mL in athletes and controls, for women who do not take hormonal contraceptives. P-values are calculated using Wilcoxon tests.

**Supplementary Table 1:** Spearman correlation between PSA and other variables among athletes and controls for women not taking hormonal contraceptives. The coloured cells indicate a Spearman correlation that is significantly different from zero with p-value < 0.01.
