# Supplementary figures and images for: Serum complexed and free prostate specific antigen levels are lower in female elite athletes in comparison to control women
Source: F1000Res. 2017 Jul 17;6:1131. [Version 1] doi: 10.12688/f1000research.11821.1 (PMC5539849; doi:10.12688/f1000research.11821.1)

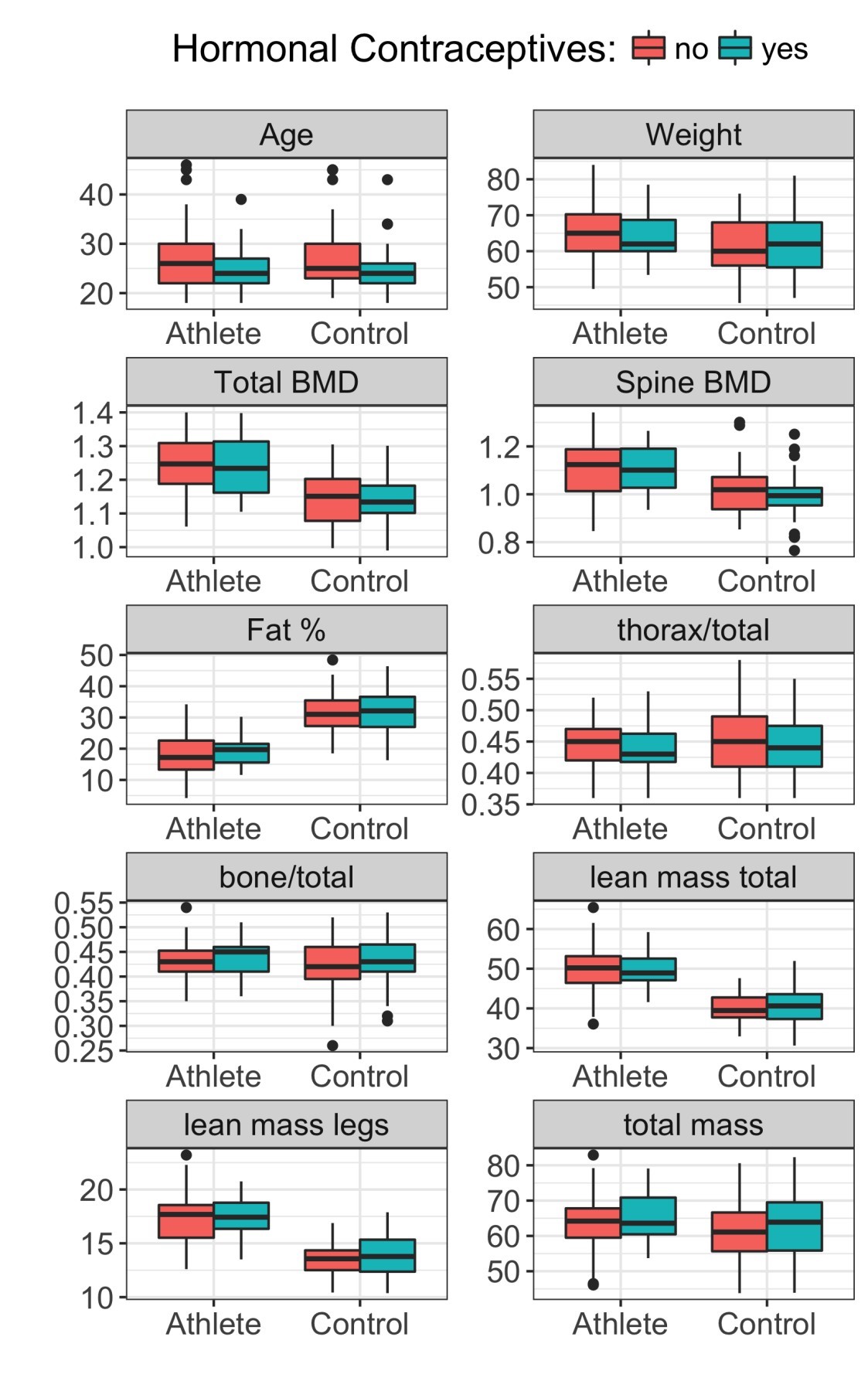

Supplement: Supplementary file 2 [file f1000research-6-12775-s0001.tgz › f3e5c27a-edb3-4f11-bf05-bd156b93f3e7.jpg]

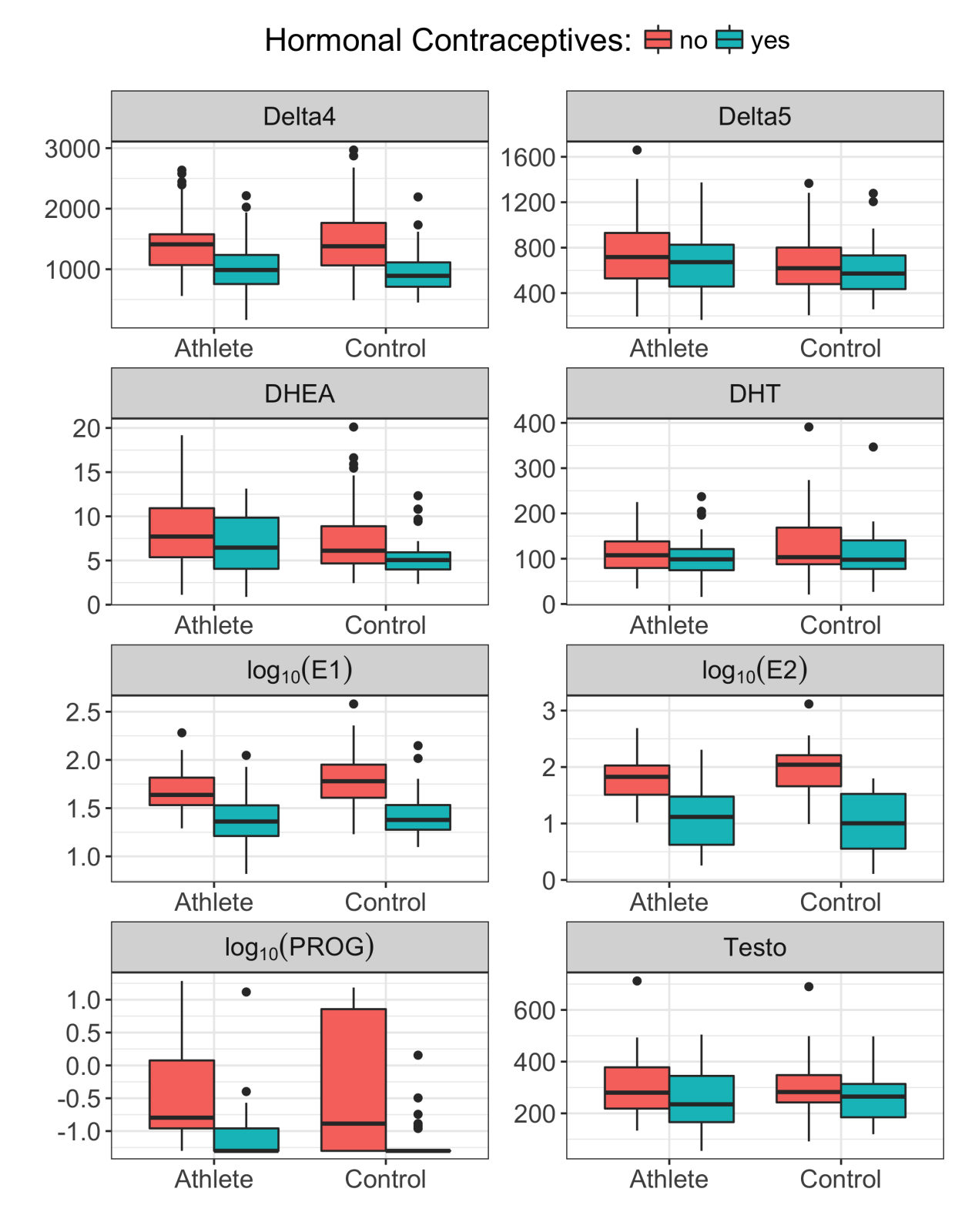

Supplement: Supplementary file 3 [file f1000research-6-12775-s0002.tgz › 35f976c5-e687-4e11-bb1d-0eae473bfef7.png]

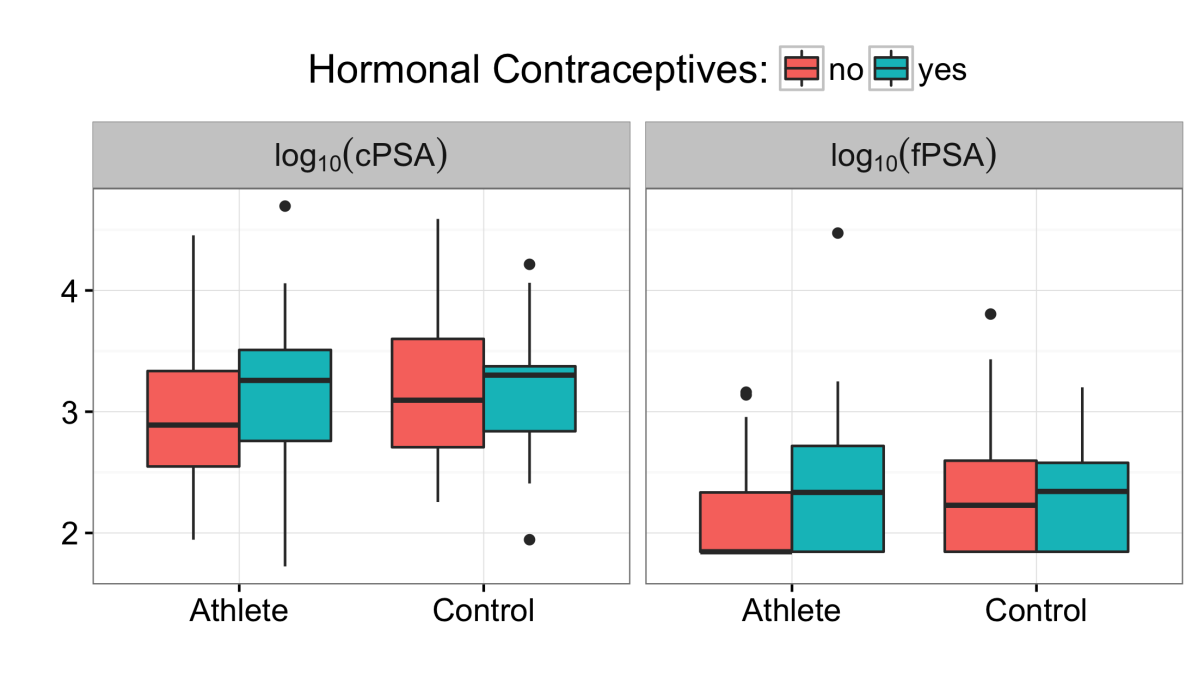

Supplement: Supplementary file 4 [file f1000research-6-12775-s0003.tgz › 7e41d8f0-b577-44ed-8bde-070239ff229c.png]

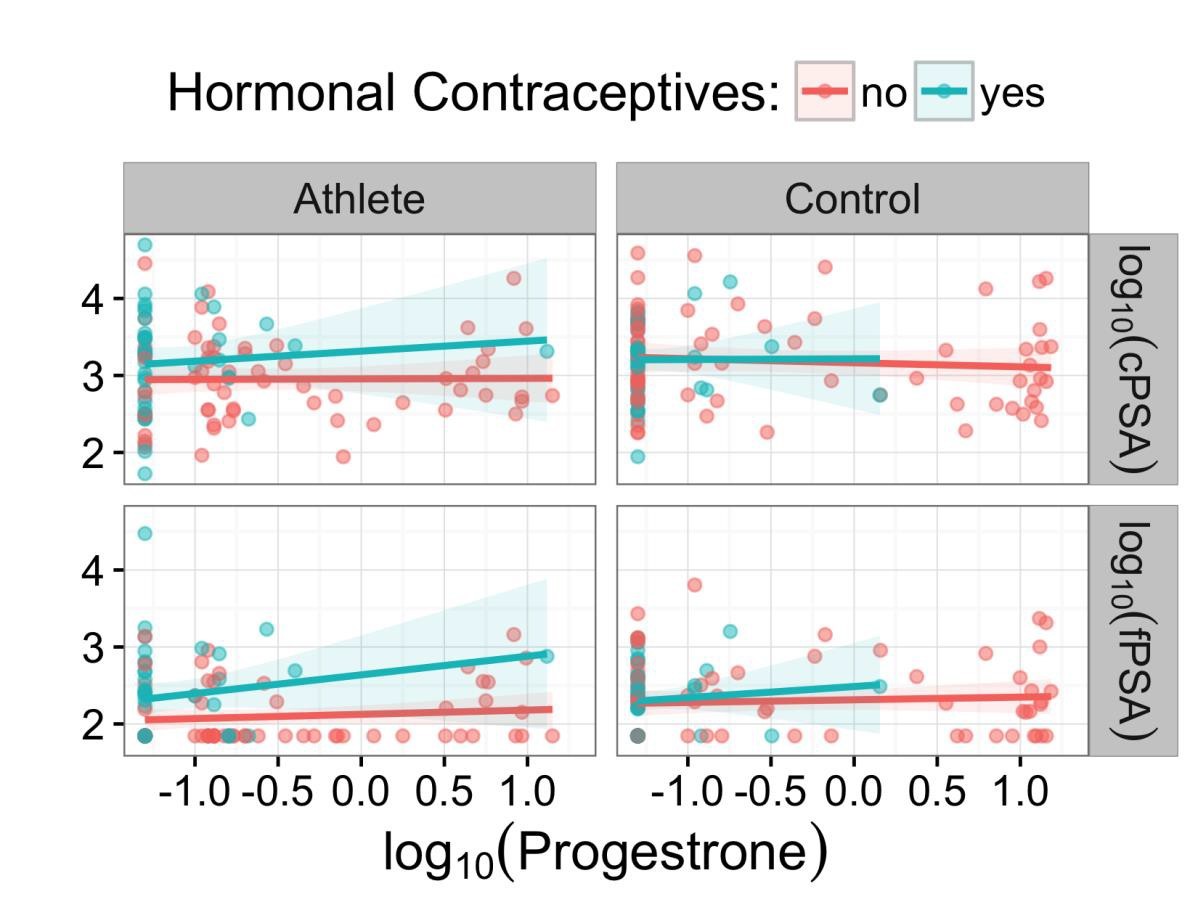

Supplement: Supplementary file 5 [file f1000research-6-12775-s0004.tgz › aa262106-1e8a-49e2-a307-df21a248045d.jpg]

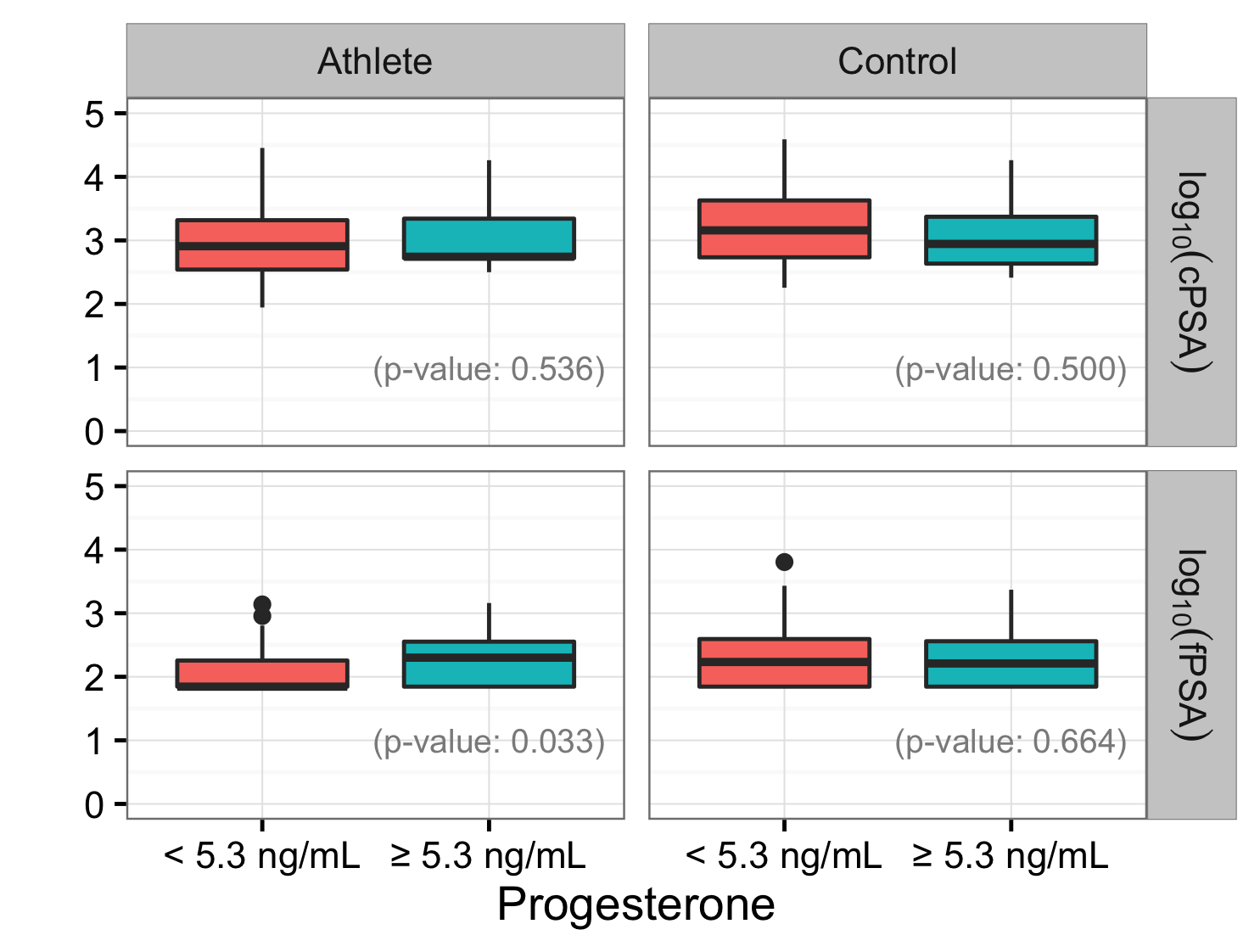

Supplement: Supplementary file 6 [file f1000research-6-12775-s0005.tgz › 6dafcf9a-9bed-49f5-b10a-375b71840bab.png]
