# Supplementary material for: Serum complexed and free prostate specific antigen levels are lower in female elite athletes in comparison to control women
Source: F1000Res. 2017 Jul 17;6:1131. [Version 1] doi: 10.12688/f1000research.11821.1 (PMC5539849; doi:10.12688/f1000research.11821.1)
Supplement: Supplementary file 7 [file f1000research-6-12775-s0006.tgz › 7fc3ca49-29b4-4bb9-9489-d014f38c0a5b.pdf]

Supplementary Table 1: Spearman correlation between PSA and other variables among athletes and controls for women not taking hormonal contraceptives. The coloured cells indicate a Spearman correlation that is significantly different from zero with p-value < 0.01.

	**Athletes**		**Controls**	
	cPSA (serum)	fPSA (serum)	cPSA (serum)	fPSA (serum)
Age (years)	0.34	0.33	-0.31	-0.09
Weight (kg)	0.11	0.04	-0.16	-0.18
Total BMD (g/cm3)	0.34	0.25	-0.10	-0.15
Spine BMD (g/cm)	0.10	0.04	-0.01	-0.10
Fat %	-0.40	-0.34	-0.13	-0.11
thorax/total (fat)	0.08	0.14	0.16	0.14
bone/total (fat)	-0.04	-0.09	-0.23	-0.20
lean mass total (kg)	0.40	0.31	-0.05	-0.12
lean mass legs (kg)	0.31	0.26	-0.01	-0.14
total mass (kg)	0.10	0.07	-0.12	-0.20
E1 (pg/mL)	0.22	0.15	0.06	0.20
E2 (pg/mL)	0.16	0.13	-0.03	0.11
DHEA (ng/mL)	0.23	0.01	0.52	0.40
Testo (pg/mL)	0.52	0.21	0.42	0.39
Delta4 (pg/mL)	0.45	0.18	0.51	0.37
Delta5(pg/mL)	0.36	0.09	0.48	0.39
DHT(pg/mL)	0.28	0.08	0.28	0.20
PROG (ng/mL)	-0.01	0.04	-0.06	0.12
