# Supplementary material for: Serum complexed and free prostate specific antigen levels are lower in female elite athletes in comparison to control women
Source: F1000Res. 2017 Jul 17;6:1131. [Version 1] doi: 10.12688/f1000research.11821.1 (PMC5539849; doi:10.12688/f1000research.11821.1)
Supplement: Peer review history

# Peer review: Referee response for version 1

**DOI:** 10.5256/f1000research.12775.r24410

**Published:** 2017-08-01

**Authors:** Geoffrey S. Baird

**Affiliations:** 1 Department of Laboratory Medicine, University of Washington, Seattle, WA, USA

The authors report on measurements of free and complexed PSA, as well as a variety of steroid hormones, in a cohort of Olympic athletes as well as a control group of sedentary women. The PSA assays used are “5 ^th^ generation” and therefore sensitive enough to detect the very low levels present in women.

  

- the fact that nearly half of the measured fPSA values are undetectable (or rather, below the limit of detection) makes me question the appropriateness of the correlation analysis in Figure 1; perhaps two correlations (one in supplemental material) that show the correlation in those with measurable fPSA would be more statistically sound?
- The finding of lower PSA in athletes is, as pointed out by the authors, not what would have been expected based on prior work. The likelihood of a confounder is thus high, in my opinion. The authors reasonably mention the role of breast PSA production as a possible confounder. However, it is unclear whether or not the experimental setup here could address an effect that was not linearly related to athletic activity. For example, there are many possible differences between Olympians and sedentary people, so perhaps a control group of non-elite athletes might better be able to address the physiologic question about PSA regulation by androgens, as there could be fewer confounding issues.
- The athletes in the study had much higher lean body mass, and the sedentary controls had more adipose mass. Does adipose tissue produce or directly up-regulate PSA?

The athletes had nearly identical testosterone as the sedentary women. Is that expected? Would it be possible to retrospectively get testosterone-epitestosterone ratios?

I have read this submission. I believe that I have an appropriate level of expertise to confirm that it is of an acceptable scientific standard.

# Peer review: Referee response for version 1

**DOI:** 10.5256/f1000research.12775.r24245

**Published:** 2017-07-26

**Authors:** Yves Courty

**Affiliations:** 1 Faculty of Medicine, INSERM U1100 (French National Institute of Health and Medical Research), Center of Respiratory Pathologies (CEPR), Tours, France

To perform their research, the authors have used recognized approaches for the analysis of free and complexed PSA, androgens, estrogens and progesterone in sera of elite athletes and control women. This manuscript is scientifically sound, very well written and informative.

 A comment on the lack of correlation between cPSA  or fPSA and progesterone would be desirable since other authors have shown that serum PSA fluctuates with the menstrual cycle.

I have read this submission. I believe that I have an appropriate level of expertise to confirm that it is of an acceptable scientific standard.
